# Implementation of Point-of-Care Diagnostics in Rural Primary Healthcare Clinics in South Africa: Perspectives of Key Stakeholders

**DOI:** 10.3390/diagnostics7010003

**Published:** 2017-01-08

**Authors:** Tivani P. Mashamba-Thompson, Ngcwalisa A. Jama, Benn Sartorius, Paul K. Drain, Rowan M. Thompson

**Affiliations:** 1 Discipline of Public Health Medicine, School of Nursing and Public Health, University of KwaZulu-Natal, Durban 4001, South Africa; Sartorius@ukzn.ac.za; 2 Discipline of Rural Health, School of Nursing and Public Health, University of KwaZulu-Natal, Durban 4001, South Africa; Jaman@ukzn.ac.za; 3 International Clinical Research Center, Department of Global Health, University of Washington, Seattle, WA 98105, USA; pkdrain@uw.edu; 4 Division of Infectious Diseases, Department of Medicine, University of Washington, Seattle, WA 98105, USA; 5 Department of Epidemiology, University of Washington, Seattle, WA 98105, USA; 6 Department of Surgery, Massachusetts General Hospital, Boston, MA 02114, USA; 7 Department of Maths, Science and Technology, Embury Institute, Durban, 4001, South Africa; rowant@eite.ac.za

**Keywords:** point-of-care diagnostics, performance, primary healthcare clinic, key stakeholders

## Abstract

Introduction: Key stakeholders’ involvement is crucial to the sustainability of quality point-of-care (POC) diagnostics services in low-and-middle income countries. The aim of this study was to explore key stakeholder perceptions on the implementation of POC diagnostics in rural primary healthcare (PHC) clinics in South Africa. Method: We conducted a qualitative study encompassing in-depth interviews with multiple key stakeholders of POC diagnostic services for rural and resource-limited PHC clinics. Interviews were digitally recorded and transcribed verbatim prior to thematic content analysis. Thematic content analysis was conducted using themes guided by the World Health Organisation (WHO) quality-ASSURED (Affordable, Sensitive, Specific, User friendly, Rapid and to enable treatment at first visit and Robust, Equipment free and Delivered to those who need it) criteria for POC diagnostic services in resource-limited settings. Results: 11 key stakeholders participated in the study. All stakeholders perceived the main advantage of POC diagnostics as enabling access to healthcare for rural patients. Stakeholders perceived the current POC diagnostic services to have an ability to meet patients’ needs, but recommended further improvement of the following areas: research on cost-effectiveness; improved quality management systems; development of affordable POC diagnostic and clinic-based monitoring and evaluation. Conclusions: Key stakeholders of POC diagnostics in rural PHC clinics in South Africa highlighted the need to assess affordability and ensure quality assurance of current services before adopting new POC diagnostics and scaling up current POC diagnostics.

## 1. Introduction

Rural communities are marginalized with regard to the distribution of healthcare services when compared to urban communities [1]. Delayed disease diagnosis, as a consequence of poor access to healthcare services, has been demonstrated as one of the major problems in rural communities [2]. Point-of-care (POC) diagnostics has the potential to improve healthcare access in settings that have limited laboratory infrastructure. Use of POC diagnostics can ensure the completion of the test and the treatment cycle in the same encounter, which has immense potential to reduce diagnostic and treatment delays and to impact patient outcomes [3,4,5,6]. Early diagnosis of infectious diseases, such as human immunodeficiency virus (HIV) and tuberculosis (TB), and rapid initiation to treatment is a key strategy to control these diseases [7]. HIV/AIDS is known as the leading cause of death in the HIV pandemic areas, contributing to between 19,000 and 56,000 maternal deaths in 2011 (6%–20% of total maternal deaths) [8]. South Africa accounts for 18% of global HIV infections, with approximately 6.7 million people infected [9]. Significant progress has been made in developing and the deployment of affordable HIV POC diagnostic tests for resource-limited settings [7,10]. However, there are still many unmet diagnostic needs for some of the well-established communicable diseases, such as tuberculosis [7]. To date, despite the high HIV disease burden, tests for management and monitoring of HIV, such as CD4 count and viral load test, as well as TB, are not offered at POC in rural and resource-limited settings in South Africa.

South Africa’s disease burden is characterized by a quadruple burden of communicable, non-communicable, perinatal and maternal and injury-related disorders, which are exacerbated by the high HIV prevalence [9,11]. Despite this high communicable disease burden in South Africa, our review revealed that South Africa offers fewer POC tests for communicable diseases than the USA, which has a low disease burn [12]. Prompt access to these new POC tests is encouraged in high disease-burdened and resource-limited settings [13]. The adoption of the United Nations Sustainable Development Goals 3 (SDG3) has the potential to prompt the increased availability of POC diagnostics in rural and resource-limited settings [14]. It has been estimated that the deployment of new POC diagnostics could prevent more than 1.2 million deaths in the low-and-middle income countries; this includes deaths caused by HIV/AIDS co-infections, such as bacterial pneumonia, syphilis, tuberculosis and deaths caused by malaria [15]. Novel diagnostics, such as mobile health technology aimed at improving healthcare access for women with gestational diabetes mellitus and to enable home-based blood glucose monitoring, are currently under development [16]. As the use of POC diagnostics expands to resource-limited settings, appropriate strategies to innovate, adapt and reduce the cost of these diagnostics need to be adopted to ensure impact on health outcomes [17].

The World Health Organisation (WHO) developed criteria for the ideal characteristics for a POC test in resource-limited settings. Successful implementation of POC diagnostics in resource-limited settings is based on its compliance with the WHO quality-ASSURED (Affordable, Sensitive, Specific, User friendly, Rapid and to enable treatment at first visit and Robust, Equipment free and Delivered to those who need it) criteria [12,13,18,19]. The entire process of POC diagnostics, from the pre-analytic through the analytic and post-analytic phases, has hidden reliability risks, which include false-positive and false-negative test results, which can lead to gross medical errors [20]. POC diagnostics, such as the oral-based OraQuick ADVANCE Rapid HIV-1/2, have been shown to identify fewer persons with HIV infection compared to fingerstick, such as the Uni-Gold Recombigen HIV test, Determine HIV-1/2 Ag/Ab Combo and INSTI HIV-1 Rapid Antibody [21]. Therefore, POC diagnostics’ quality assurance through implementation of all appropriate components of quality systems is one of the main aspects required to ensure efficacy and sustainability [22].

A study from India revealed the main challenges in the implementation of POC diagnostics to be due to relationships among providers, as well as relationships between providers of POC diagnostics and patients [23]. In addition, low-and-middle income countries’ challenges included issues relating to the following: economic; policy-related regulatory; laboratory capacity; infrastructure; quality control and quality assurance; workflow balance; training; supply chain; infection risk; administrative/operational; technical/medical; awareness; health systems-related; and cultural/societal [24]. Given the complexity of POC diagnostics and implementing them in resource-limited settings [14], careful consideration of the specific context of each setting is vital during the implementation to ensure effective utility in these settings [25]. In our previous review, we have recommended a lean and agile quality management system, which requires the involvement of POC diagnostics’ key stakeholders to ensure the sustainability of POC diagnostic services in low-and-middle income countries [26]. It has been demonstrated that the applicability and sustainability of these technologies requires the involvement of all stakeholders during implementation [27]. Despite this, there is limited evidence from the rural health service stakeholder perspective on the current performance of POC diagnostics. The purpose of this study was to explore different key stakeholders’ perceptions on the implementation of POC diagnostics in rural primary healthcare (PHC) clinics in South Africa. This exploration was conducted with reference to the relevant WHO quality-ASSURED criteria for POC diagnostics in resource-limited settings.

## 2. Materials and Methods

**Figure 1:**
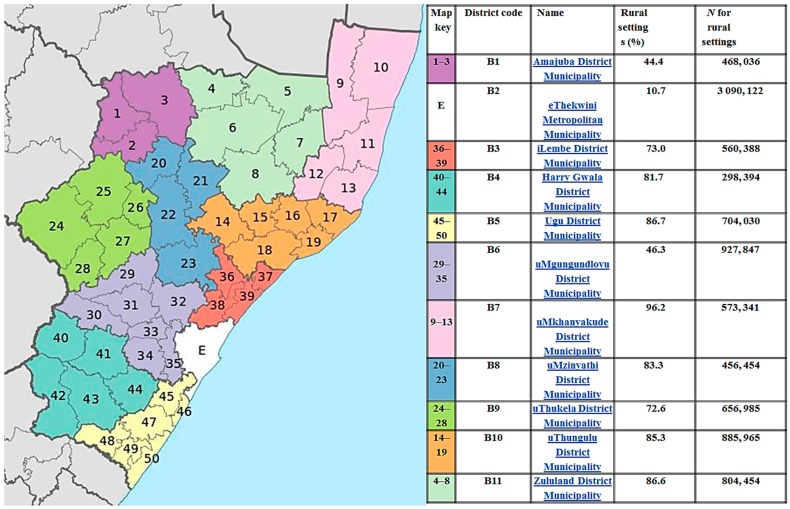
Map of KwaZulu-Natal province and percentage of rural settings in each district [29,30]. *N*, Population size.

This study was conducted as part of a large study entitled: “Evaluating the accessibility and utility of HIV-related point-of-care diagnostics for maternal health in rural South Africa: a study protocol”. The large study protocol has been published in the BMJ Open journal [28]. The large study included a survey of accessibility, availability and usage of POC diagnostics tests in 100 rural PHC clinics districts with all 11 KwaZulu-Natal (KZN) districts. KZN province is largely rural (Figure 1) [29,30]. We defined rural populations according to the South African rural development framework adopted by the South African government in 1997 as sparsely-populated areas in which people farm or depend on natural resources, including villages and small towns that are dispersed throughout these areas [31]. This included large settlements in the former homelands, created by apartheid-era removals, which depend for their survival on migratory labor and remittances [32].

### 2.1. Ethics

This study protocol was approved by the KZN Department of Health’s Ethics Committee (Approval Number HRKM 40/15, 20 March 2015) and the University of KZN Biomedical Research Ethics Committee (Approval Number BE484/14, 23 February 2015). Study participants were provided with an information sheet explaining the objectives of the study, and all participants signed informed consent forms prior to participation.

### 2.2. Study Sample and Setting

Point-of-care testing key stakeholders from rural PHC clinics that have been shown to have low POC accessibility, availability and usage from the survey were interviewed as part of the study. We defined these key stakeholders as professionals who are responsible for the implementation and the smooth running of POC diagnostics in rural PHC clinics in KwaZulu-Natal, South Africa. The following types of stakeholders were included in the study: rural PHC clinic managers; users of POC diagnostics in rural PHC clinics; public health officials involved in the implementation of POC diagnostics in rural PHC clinics; POC diagnostics developers for rural and resource-limited settings; and National Health Laboratory Service (NHLS) managers of laboratories that offered pathology testing services to the clinics.

### 2.3. Data Collection

Data for this study were collected between September 2015 and January 2016 in different settings where the stakeholders or public health officials serve including rural PHC clinics, department of health offices and laboratories. Telephonic interviews were conducted for the POC developer interviews, as they were not based in South Africa. In-depth interviews were conducted with key POC stakeholders to assess their perspective on diagnostics implementation in rural primary healthcare (PHC) clinics in South Africa. Interviews were conducted until saturation was reached: when no additional information was immerging from the interviews. All interviews were conducted in English by Tivani P Mashamba-Thompson (T.P.M.-T.) using an interview guide, which had open-ended questions (see Supplementary Material).

### 2.4. Data Entry and Analysis

The interviews conducted were audio recorded and transcribed verbatim in Microsoft Word 2013. Verbatim transcription of all interviews, with study participant’s checking to seek points of clarification in relation to issues arising from interviews, was performed to ensure the validity of the interviews. Thematic content analysis was performed by TPM-T and Rowan Mark Thompson (RMT) to increase precision by using NVIVO v10 software (QSR International Pty Ltd., Melbourne, Australia). Patterns of POC diagnostic implementation facilitators and challenges were identified from respondents’ interviews. First, participants’ responses were coded into categories, which were then grouped into themes. The WHO quality-ASSURED criteria for POC diagnostics in resource-limited settings were used as a framework for analysis. The themes were guided by the WHO quality-ASSURED criteria (Table 1). The codes were grouped into similar concepts that reflect context about local factors that determine healthcare workers and patients’ engagement with POC diagnostics. An audit trail for assessing the entire research process, from data collection to reporting, was also performed.

## 3. Results

A total of 11 interviews were conducted from POC diagnostics key stakeholders. Six participants reported playing a variety of crucial roles in the implementation of POC diagnostics in rural and resource-limited settings, and two reported no involvement (Table 2). From the stakeholders who reported involvement in POC implementation, disapproval of and resentment with the level of involvement in the implementation were reported.

District PHC manager: “*For us, there is no type of a POC that we chose. I think it’s our principals up there, in the head office because we used to get some memo that we’ll be implementing this, this is available and how are you going to get access of it. And some of them, they will come to train. How to use the test? But, as a district, we haven’t invented anything*.”

In-depth interviews were held with a total of 11 POC key. Table 2 elaborates on the role of each stakeholder that participated in this study.

All stakeholders understand POC diagnostics as a rapid testing service provided at POC, intended to inform clinical decisions. The requirement for the smooth running of PHC-based POC diagnostic services has also been outlined:

POC diagnostics developer: “*POC testing requires, first of all, patient willing to give a sample, requires someone that is working with the patient to know that they need to know that they need to be tested and someone who knows that they need to act upon the results and it also involve a support chain to get the test, people that are running the test need to be able to access the test, so distribution chain. So, it requires some training of people to be able to use the test, whether is the patient is themselves or the clinician working with the patient*.”

The study was interested in assessing the implementation of POC diagnostics in rural PHC clinics with reference to the relevant WHO quality-ASSURED criteria for POC diagnostics in resource-limited settings. Guided by the above criteria, the following core themes were identified: Theme 1: accessibility; Theme 2: effectiveness; and Theme 3: reliability. Challenges and barriers related to the above themes are discussed as sub-core themes.

### 3.1. Theme 1: Accessibility of POC Diagnostics in Rural PHC Clinics

Stakeholders reported that the main factors related to the accessibility of POC diagnostics in rural PHC clinics are the need for scaling up, supply chain management and cost effectiveness of POC diagnostics. When it comes to the issue of scaling up and optimizing the current services in order to improve the accessibility of POC diagnostics, one of the stakeholders had this to say:

POC diagnostics developer: “*The advantage of the adult test for initiation of therapy is that, again it knocks with compliance because if you measure the viral load at the first time you see the patient and you start the therapy and they come back a month later for the follow-up is that they can see that the viral load is coming down and that the drug is being effective. The advantage of monitoring a viral load after the therapy depends an awful lot on the location and whether results can get turned around in a couple of weeks*.”

Despite the reported need for POC scale-up in order to address patients’ unmet needs, other stakeholders disapproved of POC diagnostics in resource-limited settings due to challenges such as the need for staff training, cost of implementation and cost of diagnostics:

Laboratory manager: “The first challenge in terms of training and competency, we have been involved in training sessions that the department… KZN Department of Health has put together and where people are trained and given competency certificates. But in the second issue where there is external audit of somebody doing *p*-test counselling, doing the test and doing post-test counselling, that’s I think … still a gap.”

POC developer: “Paying for it, it more than doubled the cost of performing the test, where is that money coming from? And the other way to save a little bit of that money is to ask the nurses to perform that test. Nurses will take the time out and they are already overworked and underpaid, so I think the financial issues are quite a challenge.”

Stakeholders also report poor-supply-chain-induced service interruption as a barrier for accessing POC diagnostics in rural PHC clinics:

PHC clinic manager: “*But for the start of this year, although sometimes we do have the interruptions of the stock especially the rapid test for double R, for STI syphilis. We had some interruptions before. Although we do have a back-up that when there is no double R rapid test, we can send the blood specimen to the laboratory. Although, it’s costly than to do … yes. So yeah, we’re used to having those interruptions*.”

### 3.2. Theme 2: Effectiveness of POC Diagnostics in Rural PHC Clinics

Stakeholders perceive the current POC diagnostics as more effective than laboratory tests in rural PHC clinics and more convenient for patients. The effectiveness of POC diagnostics in these settings has also been compared to laboratory testing in terms of cost effectiveness, reducing loss to follow-up and enabling rapid results and early diagnosis:

Laboratory manager: “*I think the biggest advantage has been that for the patient, results are available within 15 to 20 minutes, so there is no follow-up, there is no cost incurred on the patient coming back for the result and take further tests and at the same time, the laboratory saves on cost and reduces cost and time so that expensive HIV tests are not done. Because we have noticed as the number of rapid HIV rapid test sites have grown in the province, our HIV lab testing has been decreasing over the years*.”

POC diagnostics was reported to be meeting patients’ needs with regard to disease management, improving access to healthcare, reducing the number of clinic visits and helping them save on transport costs:

POC diagnostics user: “*I think the advantages is that the patient per say does not have to come back to the facility. They only visit the facility once and they get the service at the same time in the same day. They don’t have to spend money travelling to the facility again*.”

Stakeholders provided recommendations for ensuring the effectiveness of future POC diagnostics with relevance to POC diagnostics stakeholder collaboration, research, development and quality management systems, as well as monitoring and evaluation (Table 3).

### 3.3. Theme 3: Reliability of POC Diagnostics in Rural PHC Clinics

The reliability of POC diagnostics has been reported as one of the major concerns for the current POC diagnostic services in rural and resource-limited PHC clinics. Stakeholders revealed challenges related to poor quality management, monitoring and evaluation and the environment for POC diagnostic services in rural and resource-limited settings. Stakeholders raised concerns about quality management systems employed for POC diagnostics in rural PHC clinics:

Laboratory manager: “*There has to be a correlation of the actual results in relation to the laboratory results. Second thing will be the competence of staff using it: have they been trained and are they competent in this machine? The third thing will be the controls that are being utilised, is the machine being controlled, maintained etc.? If I have to come up with POCT that I have been using, from a very limited knowledge on POCT institution, I once find that they are measuring things without being deemed competent on the instrumentation. There is a lack of control to run and maintenance and lack of standard operating procedures (SOPs) to follow on using the instrumentation*.”

A stakeholder also reported concerns on the impact of test storage on the quality of the services:

District PHC manager: “*I think the challenges that we have actually experienced when it comes to quality control per say. At times, some of the testing that we are using is of not so good quality. Or else, maybe there was problem with the storage and the facility-based testing per say being of good quality. I think it has been quality that has been the challenge*.”

## 4. Discussion

### 4.1. Main Summary of Findings

This study explored the barriers to and the facilitators for the implementation of POC diagnostics in rural and resource-limited settings. The results of the interviews demonstrated the perceived and actual accessibility, the effectiveness and reliability of POC diagnostics as it impacted on stakeholders’ decisions to develop, adopt and use these services for patient care. The majority of stakeholders expressed the need for POC diagnostics scale-up in rural PHC clinics, but raised concerns about the reliability of the current POC diagnostics and the supply chain management and staff competence for POC diagnostic services in rural PHC clinics. The stakeholders also made recommendations relating to areas that need to be improved to ensure the effectiveness of future diagnostics. The results of this study also reveal the limited involvement of laboratories in the implementation of POC diagnostics that are currently used in rural PHC clinics in South Africa.

### 4.2. Strengths and Limitations

Data saturation was reached after 11 interviews during the data collection phase. This research provides a wide overview of facilitators, barriers and challenges related to the implementation of POC diagnostics in relation to the WHO quality-ASSURED criteria for rural PHC clinics in South Africa. To the best of our knowledge, this is the first study to draw upon perspectives from a wide range of POC diagnostics key stakeholders on the implementation of POC diagnostics in rural and resource-limited PHC clinics. Exporting different stakeholder perspectives on the implementation of POC diagnostics has enabled richer understanding of influential factors that impact on decision making at different levels of POC diagnostics implementation. This study has also enabled deeper theoretical understanding of POC stakeholders’ roles in the context of POC diagnostic services in rural PHC clinics in South Africa.

Despite the importance of the data provided by this study, there are two main limitations that need to be noted. Firstly, it is important to note that the research is set within a high HIV-prevalence region [9], with limited access to laboratory infrastructure [33]. Although the study has provided important information, which relates to contextual and organizational factors that can influence decision making during the implementation of POC diagnostics in rural and resource-limited settings, these findings may not be generalized to the implementation of POC diagnostic services in a developed setting or other resource-limited settings with different characteristics from rural KZN. The other unavoidable limitation of this study was the use of telephonic interviews for two of the interviews with POC diagnostics developers. Due to the limited travelling funds for the study and the distance from the research site (University of KwaZulu-Natal, South Africa) to the POC developers’ location (United States of America), face-to-face interviews could not be conducted. Telephonic interviews are known to result in a loss of contextual and nonverbal data and to compromise rapport, probing and interpretation of responses [34]. However, the use of telephones also has advantages, and these include allowing respondents to feel relaxed and able to disclose sensitive information during the interview [34]. Therefore, this could also be interpreted as an additional strength of the study.

### 4.3. Comparison with Existing Literature

Although POC diagnostics are available for use in PHC clinics in South Africa, the majority of pathology tests are still carried out in laboratories [33]. Use of laboratory testing has been associated with artificially prolonged turnaround times, strain on human resources and quality of testing and compounding additional diagnostic and treatment delays [33]. South Africa accounts for 18% of global HIV infections, with approximately 6.7 million people infected [9]. Each day, there are almost 1000 new HIV infections, the majority of which are heterosexually transmitted [35]. Failure to control communicable diseases is, in part, due to missed opportunities to correctly diagnose and treat infectious cases [36]. Stakeholders highlighted the importance of scaling POC diagnostics in rural PHC clinics to help enable early detection of HIV and improve treatment delays, particularly to priority groups, such as HIV-infected pregnant women and babies. This supports the WHO 2013 Consolidated Antiretroviral (ARV) Guidelines and the UNAIDS “90-90-90” strategy, which advocate for continued expansion and decentralization of HIV testing, care and treatment in resource-limited settings [37].

The findings of this study support the wider literature in emphasizing the importance of addressing barriers and challenges related to the implementation of POC diagnostic services to ensure improved implementation of future diagnostics for the improvement of health outcomes in rural and resource-limited settings [13,23,24,38]. Our study findings confirm the results of an early systematic review by Jones et al. [39], which demonstrated PHC health workers’ concerns about the reliability and cost of POC diagnostics. In line with the study findings, supply chain management systems of essential health commodities have been reported as experiencing problems leading to stock out and expiry of essential reagents [40]. The importance of maintaining enhanced, interactive and highly responsive supply chain management for maintaining adequate quantities of testing stocks is essential [41]. Stakeholder recommendations on addressing barriers to POC implementation in rural PHC clinics were in line with the WHO recommendations for POC diagnostics for resource-limited settings [13,18,19] (Table 1). The results of this study show the need for human resources to ensure the quality of POC diagnostic services, and this supports the finding of a recent study in a similar setting [23].

## 5. Conclusions

The effectiveness of POC diagnostics on patient outcomes, including improving access to diagnosis and treatment, has been demonstrated [5,6,24,42,43,44,45,46,47,48]. Increasing the accessibility of POC diagnostics to resource-limited settings with poor access to laboratory infrastructure is one of the global health priorities [49]. Our findings highlight significant challenges in the implementation of POC diagnostic services in rural PHC clinics. Poor quality management systems and supply chain management and staff competency have been highlighted as the major challenges that need to be addressed before scaling-up current POC diagnostic services in rural PHCs. Measuring accuracy and reliability through sensitivity, specificity and positive and negative predictive value testing is a fundamental quality management criterion of any diagnostic test and requires laboratory involvement [50]. Therefore, there is a need for laboratory involvement in ensuring continual quality POC service delivery in these settings. Systematic strategies to improve the POC diagnostics supply chain management and staff competency for POC diagnostic services will be vital to ensure the sustainability of these services in rural and resource-limited settings. Collaborative efforts among POC stakeholders to address the above barriers will be essential in ensuring improved implementation of POC diagnostics in these settings.

## Figures and Tables

**Table 1:** The ASSURED (Affordable, Sensitive, Specific, User friendly, Rapid and to enable treatment at first visit and Robust, Equipment free and Delivered to those who need it) criteria for ideal point-of-care (POC) diagnostics for use in resource-limited settings [13,18,19].

Criteria	Characteristics
Affordable	Purchasable price for settings comprised of a population at risk of infection
Sensitive	Results contain minimal false negatives (99%)
Specific	Results contain minimal false positives (99%)
User-friendly	Required minimal steps to carry test
Rapid and robust	Short turnaround time and no need for refrigerated storage
Equipment free	No need for complex equipment
Delivered	Made accessible to end users

**Table 2:** Stakeholder involvement in POC implementation for rural primary healthcare (PHC) clinics in South Africa.

Stakeholder	Role in POC Diagnostics Implementation
3 public health officials from three KwaZulu-Natal (KZN) districts	District PHC managers responsible for deployment and overseeing POC diagnostics services in rural PHC clinics. Although the district managers are based in the district offices rather than in rural PHC clinics, they maintain regular contact with the clinics to monitor to day-to-day running of clinic services, including POC diagnostic services.
POC developers-researcher for the most accessible, available and used POC test in rural South Africa	Research and development of HIV rapid test. This stakeholder was part of the team of scientists that were involved in the development and deployment of HIV rapid tests in South Africa. Although the stakeholder is based in the USA, the stakeholder still plays a significant role in the implementation of HIV-related POC diagnostics in South Africa.
POC developer-manager for all POC diagnostics for resource-limited settings	Managing research, development and deployment of POC diagnostics to resource-limited settings. Although this stakeholder is based in the USA, the stakeholder is responsible for coordinating the development and deployment of POC diagnostics in resource-limited settings.
2 POC diagnostics users based on rural PHC clinics	Patient testing in rural PHC clinics. In additions these stakeholders are responsible to maintaining the supply chain management for POC diagnostics services from the clinic side.
2 Clinic managers for rural PHC clinics	Managing the smooth running of POC diagnostic services in POC clinics. Clinic managers are responsible for managing POC diagnostics users in the clinic and for reporting to the district managers.
2 NHLS managers from KwaZulu-Natal	Not directly involved in the PHC clinic POC diagnostics implementation, but involved in laboratory-based pathology service provision. All discordant POC test results require a follow up with laboratory testing. Therefore, we perceive laboratories as one of the key stakeholders of these services.

PHC, primary healthcare; POC, point-of-care; NHLS, National Health Laboratory Services.

**Table 3:** Stakeholders’ recommendations for improving the effectiveness of POC diagnostic services in rural PHC clinics.

Relevance	Recommendations	Alignment with the WHO Quality ASSURED Criteria
Research	POC diagnostics developer: “*What you should really ask to be done or maybe to it yourself, is go do a proper outcome study with the economics involved, identify all the costs, work out all the* *labour* *and workflow issues, follow the patient to see if anything gets better and only then start to recommend that point-of-care could be done…*”	Affordability
POC diagnostics development	POC diagnostics developer: “*It is just a trade-off there, if cost is really that critical, we could design a system that is really cheap, you could do two at the time, five at a time or 10 at a time and you could share some of the common things such as a screen, a heater you only need one instead of two for two machines but the workflow is going to be impacted*.”	Affordability
Stakeholder collaboration	POC diagnostics developer: “*So, if we can have better communication between those who are on these data and the developers, I think that we can really make systems more cost effective*.” Laboratory manager: “*There must be collaboration between the departments, I mean among the whole stakeholders whenever the point-of-care testing is introduced. So that it can be validated before it will be implemented because most of the time they just implement it and then when there is a problem… imagine now you bought a number of machines, only to find that those machines, they are not producing what they are supposed to produce*.”	Sensitivity and Specificity
Quality Management	Laboratory manager: “*Like I said, provided it is done, and we are getting true results, if facility chooses, the only reason I can agree with point-of-care testing to be done in the facility, is when you need urgent results. If you look at lab machines there are internal quality controls that are run to ensure accuracy of the results to make sure that all instrumentation is performing well. Regular maintenance is done in labs by competent staff*.”	Sensitivity and Specificity
Monitoring and evaluation	PHC manager: “*I think if it can be closely monitored and evaluated and challenges can be closely addressed, I don’t think it will be a serious problem*.”	Sensitivity and Specificity

## Supplementary Materials

The following are available online at http://www.mdpi.com/2075-4418/7/1/3/s1. Interview guides for the in-depth interviews with different groups of key stakeholders.
