# Supplementary material for: Implementation of Point-of-Care Diagnostics in Rural Primary Healthcare Clinics in South Africa: Perspectives of Key Stakeholders
Source: Diagnostics (Basel). 2017 Jan 8;7(1):3. doi: 10.3390/diagnostics7010003 (PMC5373012; doi:10.3390/diagnostics7010003)
Supplement: Supplementary file 1 [file diagnostics-07-00003-s001.pdf]

Supplementary Materials: Implementation of Point-of-Care Diagnostics in Rural Primary Healthcare Clinics in South Africa: Perspectives of Key Stakeholders

Tivani P. Mashamba-Thompson, Ngcwalisa A. Jama, Benn Sartorius, Paul K. Drain and Rowan M. Thompson

Stakeholder Interview Guides

University of KwaZulu Natal, Durban, Department of Public Health Medicine

Title: Evaluating the accessibility and utility of HIV-related point of care (POC) diagnostics for maternal health in rural South Africa

Maternal health clinic healthcare worker in-depth interview

**District code:**	**Clinic category code:**
**District name:**	**Clinic name:**
**Interviewer's name:**	**Date:**

1. What is your understanding of POC testing?
2. What does POC testing involve?
3. Who are the role players in POC diagnostics services?
4. What is your role in POC testing services?
5. What are the advantages of using POC diagnostic service to you?
6. What are the challenges of using the current POC diagnostic service to you?
7. How did you overcome these challenges
8. What are the advantages of POC diagnostic services to mothers?
9. What are the challenges of POC diagnostic services to mothers?
10. How did you overcome these challenges?
11. What improvement would you like to see in the current POC diagnostics services in your clinic?
12. How do you feel about scaling up of the current POC tests in your clinic?
13. What are the anticipated advantages will POC diagnostic service scaling up have to you?
14. What are the anticipated challenges of POC diagnostic service scale up to you?
15. How did you overcome these challenges?

16. What are the anticipated advantages of POC diagnostic services scale up to mothers?
17. What are the anticipated challenges of POC diagnostic services scale up to mothers?
18. How did you overcome these challenges?
19. Is there any other information that you would like to add to this?

University of KwaZulu Natal, Durban, Department of Public Health Medicine

Title: Evaluating the accessibility and utility of HIV-related point of care (POC) diagnostics for maternal health in rural South Africa

KZN district public health official in-depth interview

**District code:**	**Clinic category code:**
**District name:**	**Clinic name:**
**Interviewer's name:**	**Date:**

1. What is your understanding of POC testing?
2. What does POC testing involve?
3. Who are the role players in POC diagnostics services?
4. What is your role in POC testing services?
5. What are the advantages of the current POC diagnostic service to your district?
6. What are the challenges of the current POC diagnostic service to your district?
7. How will you overcome these challenges?
8. What are the advantages of POC diagnostic services to the mothers your district?
9. What are the challenges of POC diagnostic services to the mothers in your district?
10. How will you overcome these challenges?
11. Is the current POC diagnostic service in district maternal clinic meeting patient's needs?
12. What improvement would you like to see in the current POC diagnostics services in the district maternal health clinics?
13. Were you involved in the implementation of POC diagnostics implementation in the district maternal health clinics?
14. What challenges did you encounter during the implementation?
15. How did you overcome these challenges?
16. Why was the current type/types of POC diagnostic chosen for this district?

17. How do you feel about scaling up of the current POC tests in your district maternal health clinic?
18. What are the anticipated advantages will POC diagnostic service scaling up have to your district?
19. What are the anticipated challenges of POC diagnostic service scale up to your district?
20. How would you district overcome these challenges?
21. What are the anticipated advantages of POC diagnostic services scale up to mothers in your district?
22. What are the anticipated challenges of POC diagnostic services scale up to mothers in your district?
23. How would you district overcome these challenges?
24. Is there any other information that you would like to add to this?

University of KwaZulu Natal, Durban, Department of Public Health Medicine

Title: Evaluating the accessibility and utility of HIV-related point of care (POC) diagnostics for maternal health in rural South Africa

Maternal health clinic Manager in-depth interview

**District code:**	**Clinic category code:**
**District name:**	**Clinic name:**
**Interviewer's name:**	**Date:**

1. What is your understanding of POC testing?
2. What does POC testing involve?
3. Who are the role players in POC diagnostics services?
4. What is your role in POC testing services?
5. What are the advantages of POC diagnostic service to you?
6. What are the challenges of POC diagnostic service to you?
7. How do you overcome these challenges
8. What are the advantages of POC diagnostic services to mothers?
9. What are the challenges of POC diagnostic services to mothers?
10. How do they overcome these challenges?

11. Is the current POC diagnostic service meeting patient's needs? Explore
12. What improvement would you like to see in the current POC diagnostics services in your clinic?
13. Where you involved in the implementation of the current POC diagnostic services in this clinic?
14. What challenges did you encounter during the implementation?
15. How did you overcome these challenges?
16. Why was the current POC diagnostic service chosen for this clinic?
17. How do you feel about scaling up of the current POC tests in your clinic?
18. What are the anticipated advantages will POC diagnostic service scaling up have to you?
19. What are the anticipated challenges of POC diagnostic service scale up to you?
20. How would you overcome these challenges?
21. What are the anticipated advantages of POC diagnostic services scale up to mothers?
22. What are the anticipated challenges of POC diagnostic services scale up to mothers?
23. How would you overcome these challenges?
24. Is there any other information that you would like to add to this?

University of KwaZulu Natal, Durban, Department of Public Health Medicine

Title: Evaluating the accessibility and utility of HIV-related point of care (POC) diagnostics for maternal health in rural South Africa

HIV and Maternal Health-related POC diagnostic developer in-depth interview

**District code:**	**Clinic category code:**
**District name:**	**Clinic name:**
**Interviewer's name:**	**Date:**

1. What is your understanding of POC testing?
2. What does POC testing involve?
3. Who are the role players in POC diagnostics services?
4. What is your role in POC testing services?
5. What are the advantages of POC diagnostic service to you?
6. What are the challenges of POC diagnostic service to you?

7. How did you overcome these challenges
8. What are the advantages of POC diagnostic services to mothers?
9. What are the challenges of POC diagnostic services to mothers?
10. How did you overcome these challenges?
11. What improvement would you like to see in the current POC diagnostics services in your clinic?
12. How do you feel about scaling up of the current POC tests in your clinic?
13. What are the anticipated advantages will POC diagnostic service scaling up have to you?
14. What are the anticipated challenges of POC diagnostic service scale up to you?
15. How did you overcome these challenges?
16. What are the anticipated advantages of POC diagnostic services scale up to mothers?
17. What are the anticipated challenges of POC diagnostic services scale up to mothers?
18. How did you overcome these challenges?
19. Is there any other information that you would like to add to this?

University of KwaZulu Natal, Durban, Department of Public Health Medicine

Title: Evaluating the accessibility and utility of HIV-related point of care (POC) diagnostics for maternal health in rural South Africa

KZN district maternal health clinic pathology service provider in-depth interview

**District code:**	**Clinic category code:**
**District name:**	**Clinic code:**
**Interviewer's name:**	**Date:**

1. What is your understanding of POC testing?
2. What does POC testing involve?
3. Who are the role players in POC diagnostics services?
4. What is your role in POC testing services?
5. What are the advantages of POC diagnostic service to your laboratory services?
6. What are the challenges of POC diagnostic service to your laboratory services?
7. How did you overcome these challenges?
8. How do you feel about scaling up of the current POC tests in your clinic?

9. What are the anticipated advantages that POC diagnostic service scaling up will bring to your laboratory?
10. What are the anticipated challenges of POC diagnostic service scale up to your laboratory?
11. How will you overcome these challenges?
12. Is there any other information that you would like to add to this.
